# Thermoelectric Cooling Performance Enhancement in BiSeTe Alloy by Microstructure Modulation via Hot Extrusion

**DOI:** 10.1002/smsc.202300245

**Published:** 2023-12-27

**Authors:** Yu Zhang, Guang Xu, Amin Nozariasbmarz, Wenjie Li, Lavanya Raman, Congcong Xing, Shweta Sharma, Na Liu, Subrata Ghosh, Giri Joshi, Mohan Sanghadasa, Priya Shashank, Bed Poudel

**Affiliations:** ^1^ Department of Materials Science and Engineering Pennsylvania State University University Park PA 16802 USA; ^2^ Nanohmics Inc. 6201 E Oltorf St Austin TX 78741 USA; ^3^ U.S. Army Combat Capabilities Development Command Aviation & Missile Center Redstone Arsenal AL 35898 USA

**Keywords:** annealing, bismuth telluride, hot extrusion, power factor, thermoelectric device

## Abstract

The demand for high‐performance materials in thermoelectric (TE) technology has driven continuous efforts to enhance the performance of commercialized Bi_2_Te_3_‐based thermoelectric materials. Here, we report success in achieving significant performance improvements in n‐type Bi_2_Te_2.8_Se_0.2_S_0.01_ through the implementation of a hot extrusion manufacturing process. This tailored manufacturing process has yielded a desired microstructure characterized by grain growth and preferred orientations. The resulting enlarged grain‐based microstructure exhibits reduced dislocations and defects that originated from plastic deformation during extrusion and post annealing. As such, the charge carrier mobility is significantly enhanced, leading to an ultrahigh power factor of approximately 51 μW cm^−1^ K^−2^ at ambient temperature. Consequently, a maximum figure of merit (zT) of 1.12 is achieved at 348 K in the combination of extrusion and annealing procedures. Using the synthesized n‐type Bi_2_Te_2.8_Se_0.2_S_0.01_ material, full‐scale cooling modules have been fabricated. These modules demonstrate record cooling performance, with a maximum temperature difference (Δ*T*) of 73.9 K at a hot‐side temperature of 300 K and a maximum cooling power density of 2.2 W cm^−2^. The cooling performance of these TE devices surpasses that of commercially available devices, establishing their potential for next‐generation TE cooling applications.

## Graphical abstract

The breakthrough in enhancing n‐type Bi_2_Te_2.8_Se_0.2_S_0.01_'s performance involves a specialized hot extrusion method, resulting in an optimal microstructure marked by grain growth and preferred orientations. This tailored manufacturing approach, combined with annealing, attains a remarkable zT of 1.12. The cooling modules exhibit unprecedented cooling performance, showcasing a record temperature difference of 73.9 K.

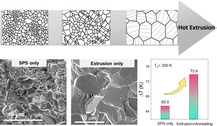

## 1 Introduction

Thermoelectric (TE) materials have garnered significant attention due to their ability to directly convert thermal energy into electrical energy and vice‐versa. TE's have been extensively studied for waste‐heat‐energy harvesting and solid‐state cooling. The performance of TE materials is typically evaluated using the dimensionless figure‐of‐merit, denoted as zT, which is defined as zT = *S* ^2^ *σT*/*κ*. Here, *S*, *σ*, *κ*, and *T* represent the Seebeck coefficient, electrical conductivity, thermal conductivity, and absolute temperature, respectively.^[^ ^1^, ^2^ ^]^

TE coolers provide extremely fast and accurate temperature control, operating without vibration and moving parts. In the realm of TE cooling applications, two crucial factors determine the cooling performance: the maximum temperature difference (Δ*T*) and the maximum cooling density achievable at zero Δ*T*, depending on the specific requirements of the intended application.^[^ ^3^ ^]^ Over the past six decades, Bi_2_Te_3_‐based alloys have been established as the leading candidates for achieving the highest Δ*T* due to their exceptional zT values near room temperature.^[^ ^4^, ^5^, ^6^ ^]^ However, the exploration of optimum cooling density is an area that remains relatively unexplored. In many TE applications where precise temperature control with small Δ*T* is paramount, such as in electronics temperature regulation and particularly in cooling hot spots,^[^ ^7^, ^8^ ^]^ the maximum cooling density under these conditions assumes greater significance. This is especially evident in high‐power electronics thermal management, where a substantial amount of heat is generated during operation, necessitating strict temperature maintenance within a narrow range to ensure optimal performance and longevity. Along this direction, the maximum heat‐flux pumping capability (*Q*_Cmax_) extracted by a TE cooling device is given as^[^ ^9^, ^10^ ^]^

(1) $$Q_{\text{Cmax}}=\left(\frac{\text{PF}\times T_{C}^{2}}{2\Delta T}-\;\kappa \right)\Delta T$$

here, PF represents the power factor of materials, *T*_C_ denotes the temperature on the cold side, and *κ* represents the thermal conductivity. The equation highlights the key factors for achieving a high *Q*_Cmax_, namely a large power factor (PF) and a small *κ*. This is particularly important when operating under small temperature differences (Δ*T*), e.g., laser diode cooling. In such scenarios, the maximization of the power factor becomes essential. Significant advancements in zT have been made over the past two decades, primarily attributed to the discovery of new materials and the implementation of innovative strategies to reduce *κ*.^[^ ^11^, ^12^, ^13^, ^14^, ^15^ ^]^ However, the thermal conductivity of representative TE systems is rapidly approaching the lower limit set by amorphous materials.^[^ ^16^ ^]^ In contrast, the power factor does not have a theoretical upper bound, yet only a few strategies have been developed to enhance its performance.

On the other hand, while Bi_2_Te_3_‐based compounds serve as representative materials for near‐ambient‐temperature TE applications, several challenges remain for this important class of materials. Specifically, n‐type Bi_2_Te_3_‐based materials exhibit inferior performance compared to their p‐type counterparts in both PF and *zT*, leading to a significant performance imbalance between the two types.^[^ ^17^, ^18^, ^19^ ^]^ The electrical transport properties of n‐type Bi_2_Te_3_ are often severely affected by mechanical nanostructuring, unlike p‐type Bi_2_Te_3_. Additionally, many high‐performance n‐type Bi_2_Te_3_‐based materials are alloyed with Se to form Bi_2_Se_*x*_Te_3−*x*_, which reduces *κ*_lat_ but sacrifices the power factor. Consequently, they typically exhibit a lower PF than their p‐type counterparts.^[^ ^20^, ^21^, ^22^ ^]^

The improvement of the PF in n‐type Bi_2_Te_3_‐based materials has been relatively limited.^[^ ^19^, ^23^, ^24^ ^]^ One notable approach that has been extensively investigated is the incorporation of Cu atoms into Bi_2_Se_*x*_Te_3−*x*_. Cu serves as an amphoteric dopant in Bi_2_Se_*x*_Te_3−*x*_, where it can either substitute for Bi atoms, inducing p‐type conduction,^[^ ^25^, ^26^ ^]^ or occupy interlayer and/or interstitial sites, resulting in n‐type conduction.^[^ ^22^ ^]^ For the latter case, significant progress has been achieved in ball‐milled and hot‐pressed Cu_0.01_Bi_2_Te_2.7_Se_0.3_ samples, which exhibited a high PF of approximately 31.5 μW cm^−1^ K^−2^.^[^ ^22^ ^]^ Recently, an extraordinary off‐stoichiometric bulk material, K_0.06_Bi_2_Te_3.18_, was successfully stabilized using a combined process involving kinetically controlled nano synthesis and subsequent spark plasma sintering (SPS). This material demonstrated exceptional performance with a very high PF of approximately 43 μW cm^−1^ K^−2^ and a zT exceeding 1.1 at 323 K.^[^ ^27^ ^]^

Here, we present a novel n‐type polycrystalline Bi_2_Te_3_ system with a nominal composition of Bi_2_Te_2.8_Se_0.2_S_0.01_, which exhibits an ultrahigh power factor (51 μW cm^−1^ K^−2^) and improved carrier mobility (150 cm^2^ V^−1^s^−1^) near room temperature. This remarkable performance is achieved through the implementation of a hot extrusion manufacturing process. This tailored manufacturing method results in a desired microstructure characterized by the growth of larger grains with preferred orientations. These enlarged grains contribute to high carrier mobility, thereby, modulating the electrical transport properties. Further, the development of a homogeneous microstructure with fewer dislocations and reduced defect density, arising from the plastic deformation occurring during extrusion, contributes to an ultrahigh power factor. We successfully fabricated 18‐pair full‐scale cooling modules based on the optimized n‐type materials developed in this study, Bi_2_Te_2.8_Se_0.2_S_0.01_. These modules demonstrated remarkable performance, with a Δ*T* of 73.9 K at a hot‐side temperature of 300 K and a maximum cooling power density of 2.2 W cm^−2^, representing the highest reported performance to date among single stage Bi_2_Te_3_‐based devices.

## 2 Results and Discussions

**Figure 1:**
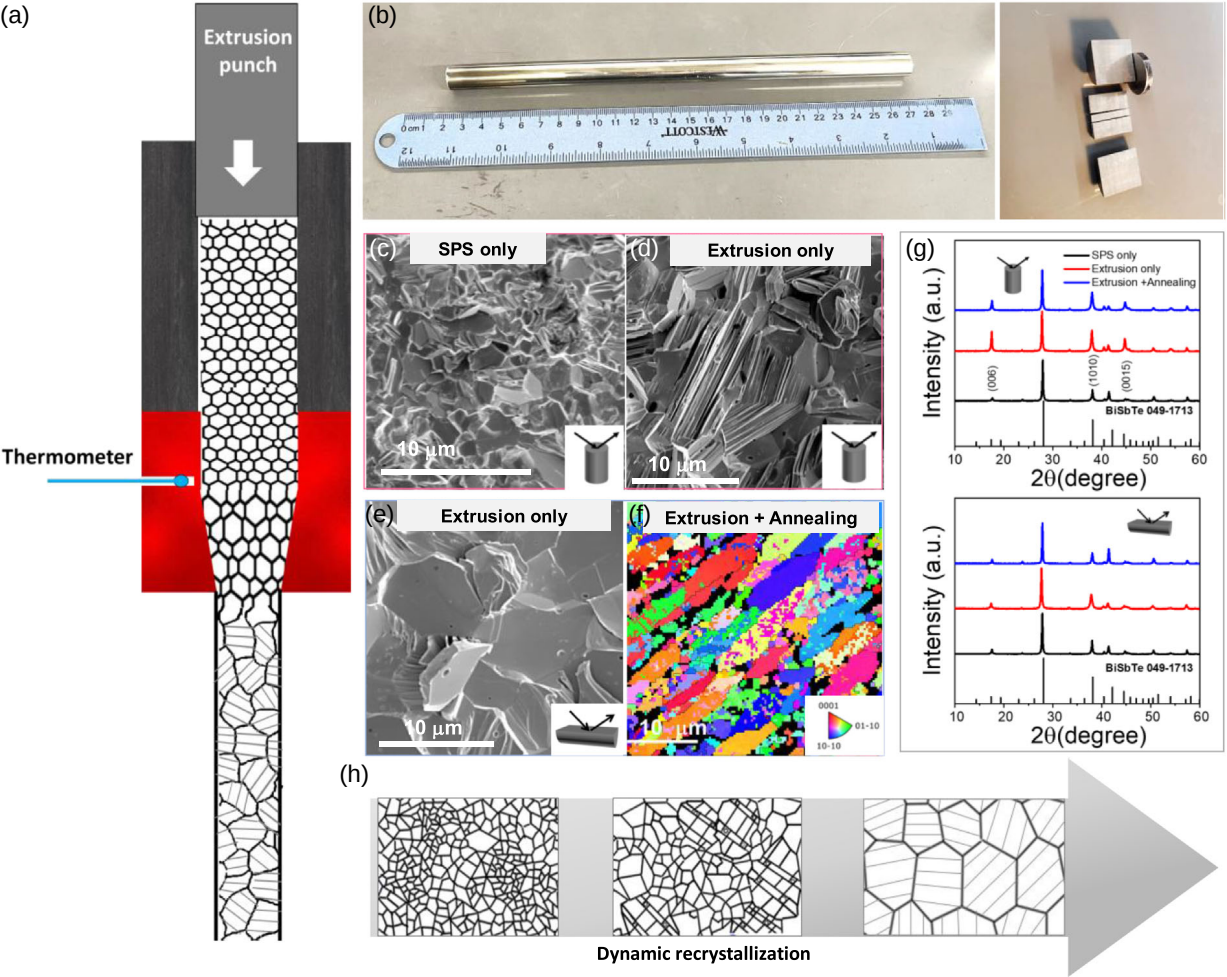
a) Schematic illustration of particle deformation and grain growth via high‐temperature hot extrusion. b) Photograph of the obtained ingot after extrusion. Representative SEM micrograph of the grain structure of Bi_2_Te_2.8_Se_0.2_S_0.01_ samples prepared by SPS only c) and extrusion from d) top‐view and e) cross‐section view, respectively. f) Electron backscatter diffraction (EBSD) images of (extrusion + annealing) samples. g) XRD patterns of consolidated pellets’ surface placed perpendicular to press/extrusion axis (up, top‐view) and parallel to it (bottom, cross‐section). h) Grain refinement in hot extrusion process.

The hot extrusion technique was employed to process ball milled Bi_2_Te_2.8_Se_0.2_S_0.01_ powder using a metal die at temperatures ranging from 325 °C to 450 °C (**Figure** **1**a,b and S1, Supporting Information). The microstructure of the extruded Bi_2_Te_2.8_Se_0.2_S_0.01_ alloys was found to be sensitive and strongly dependent on the consolidation process parameters. A comparison of grain sizes obtained from SEM microstructure analysis is presented in Figure 1c. The average grain size of the sample that underwent only SPS was determined to be approximately 2 μm (Figure S2, Supporting Information), consistent with a previous report under similar sintering conditions.^[^ ^28^ ^]^ The grains exhibited a random distribution without any noticeable crystallographic orientation or textured degree, as further confirmed by X‐ray diffraction (XRD) analysis (Figure 1g).

After the sample underwent extrusion, the grain size increased significantly, reaching up to approximately 20 μm (Figure 1d and S3, Supporting Information). These enlarged grains are believed to result from dynamic recrystallization occurring during hot extrusion where the small grains grew into large and oriented grains under elevated temperature and pressure.^[^ ^29^, ^30^ ^]^ The microstructure shows that the grain size is much larger than the original powder size, and a substantial laminar structure was formed concurrently (Figure 1d). Furthermore, as high extrusion temperatures and a slow extrusion speed were employed in the experiments, the temperature of the specimen remained sufficiently high after exiting the die to allow for additional grain growth.

XRD analysis revealed diffraction peaks corresponding to the Bi_2_Te_3_ rhombohedral phase (PDF #49‐1713) in all samples. It is observed that the (00*l*) peaks vary in polycrystalline samples with different preparation processes. To evaluate the preferential orientation along the (00*l*) plane in bulk materials, *F* factors were calculated with Lotgering method^[^ ^31^, ^32^, ^33^ ^]^: *F =* (*P−P*_0_)/(*1−P*_0_), where *P* refers to the ratio of the integral intensities of the (*00l*) planes to the intensities of the (*hkl*) planes in anisotropic samples and *P*_0_ is the ratio of the integral intensities of the (*00l*) planes to the intensities of the (*hkl*) planes in randomly oriented material. *I* and *I*_0_ are the intensities of the diffraction reflections of the measured samples and the standard rhombohedral Bi_2_Te_3_ (PDF #49‐1713), respectively. The calculated value of *F* increases significantly from 0.28 for the SPS sample to 0.36 for the extruded samples, demonstrating that the extrusion process has the effect of promoting the preferential alignment of the basal plane in the extrusion direction. These results are consistent with previous findings where it was observed that the anisotropy of Bi_2_Te_3_ can be altered after extrusion^[^ ^30^, ^34^ ^]^ or doping with impurities.^[^ ^31^ ^]^ Here, since the hot extrusion technique demonstrated the capability to control grain size and induce preferential orientation, it further addresses the influence of manufacturing process parameters on the microstructure and crystallographic orientation of n‐type Bi_2_Te_3_‐based TE materials. In addition, it has been extensively reported that rapid thermal annealing is found to be very effective in improving the thermoelectric properties of Bi_2_Te_3._^[^ ^35^, ^36^, ^37^ ^]^ After extrusion, we carried out a rapid thermal treatment on extruded rods in range of 400–520 °C for 10 min under Ar atmosphere. This will induce additional structural modifications which potentially impact the TE properties of the material. Thus, investigations on the relationship between microstructure, orientation, and TE performance were conducted and are discussed later.

**Figure 2:**
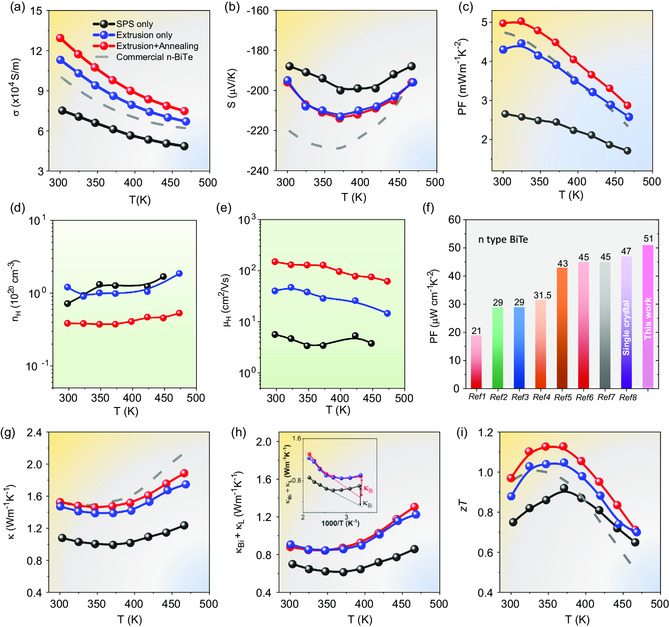
TE properties of Bi_2_Te_2.8_Se_0.2_S_0.01_. Temperature dependence of a) electric conductivity (*σ*) and b) Seebeck coefficient (*S*). c) Power factors (PF), d) Hall charge carrier concentration (*n*_H_) and e) carrier mobility (*μ*_H_). f) Room temperature PF comparison of state‐of‐art n‐type Bi_2_Te_3_ materials.^[^ ^20^, ^22^, ^29^, ^44^, ^51^, ^52^, ^53^, ^54^ ^]^ g) Thermal conductivity (*κ*). h) Lattice thermal conductivity (*κ*). The inset shows the plot of 1000/T‐dependent *κ*_L_. i) TE figure of merit (zT), with all measurement performed parallel to press axis.

The extruded rod, utilizing n‐type Bi_2_Te_2.8_Se_0.2_S_0.01_ powder, is subjected to dicing and slicing processes to produce bar and disk samples, as depicted in Figure 1b and S5, Supporting Information, for precise characterization of TE properties. The dicing/slicing direction is carefully chosen to ensure consistent measurements in the same direction as the applied force during extrusion. The temperature‐dependent TE properties are systematically investigated in the range from room temperature to 200 °C. **Figure** **2** summarizes the TE properties of Bi_2_Te_2.8_Se_0.2_S_0.01_ samples fabricated using different processing methods.

Figure 2a illustrates that the extruded sample displays significantly higher electrical conductivity (*σ*) compared to the SPS sample. Further, the combination of extrusion and annealing exhibits a notable enhancement in σ due to improved electrons mobility which will be discussed in the later section. Importantly, the enhancement in electrical conductivity is particularly prominent at low temperatures. In general, the electrical conductivity of all samples monotonically decreases with increasing temperature, indicating a degradation of semiconductor behavior. The negative Seebeck coefficient, measured for all bulk samples, confirms their n‐type conductivity with electrons as the dominant carriers. The temperature‐dependent variation of the Seebeck coefficient reveals an initial decreasing trend followed by an increasing trend beyond 370 K, suggesting bipolar conduction behavior. This behavior typically arises when the minority carriers, in this case, holes, become activated and contribute to the overall conduction.

Interestingly, despite the higher *σ* observed in the extruded samples, the absolute values of the Seebeck coefficient do not decrease but rather exhibit apparent increases, from −188 to −196 μV K^−1^ at 300 K, after extrusion and annealing process, as depicted in Figure 2b. This contrasting behavior, where both the Seebeck coefficient and electrical conductivity increase simultaneously, calls for further investigation since the improvement in σ typically accompanies with a decrease in the Seebeck coefficient. To elucidate this unusual simultaneous enhancement, hall measurements are conducted subsequently to evaluate the carrier concentration (*n*_H_) and mobility (*μ*_H_).

Figure 2d,e depict the temperature‐dependent characteristics of *n*_H_ and *μ*_H_ for samples prepared using three distinct methodologies. At ambient temperature, the extruded sample demonstrates a superior carrier mobility of approximately 40 cm^2^ V^−1^s^−1^ in comparison to the SPS sample, which exhibited a lower mobility of 7 cm^2^ V^−1^s^−1^ in the presence of the smaller weakly oriented grains due to the well‐known sensitivity of Bi_2_Te_3−*x*_Se_*x*_'s carrier mobility to the average grain size.^[^ ^38^, ^39^ ^]^ The pronounced decrease in grain boundary scattering serves as the primary factor responsible for the enhanced charge carrier transport in the extruded sample.

Many studies have revealed that annealing process for n‐type Bi_2_Te_3_ can easily decrease donor‐like defects, such as antisite defects ${\text{Bi}}_{\text{Te}}^{'}$ and vacancies $V_{\text{Te}}^{..}$,^[^ ^40^ ^]^ thus providing lower concentration of n‐type carriers, thereby enhancing the power factor and diminishing the lattice thermal conductivity of the material. To verify the behavior of donor‐like defects, we compared the *n*_H_ of samples before and after annealing. It was found that *n*_H_ drops from 1.1 × 10^20^ to 0.4 × 10^20^ cm^−3^ after annealing. Meanwhile, the combined extrusion and annealing procedure resulted in a significant augmentation of charge carrier mobility, increasing from 40.4 cm^2^ V^−1^s^−1^ in extruded sample to 150 cm^2^ V^−1^s^−1^ at ambient temperature, which is comparable to those prepared by melting techniques.^[^ ^23^, ^41^ ^]^ This notable improvement in charge carrier transport can be primarily ascribed to the reduction in the density of point defects.

In n‐type Bi_2_Te_3_, the presence of defects, such as antisite defects ${\text{Bi}}_{\text{Te}}^{'}$ and vacancies $V_{\text{Te}}^{..}$,^[^ ^40^ ^]^ significantly influences the scattering of charge carriers through electron‐defect interactions, thereby leading to an augmented carrier mobility. This assertion is concurrently supported by the observed decrease in carrier density, as depicted in Figure 2d, which can be attributed to a reduced defect density resulting from the annealing process.^[^ ^42^, ^43^ ^]^ Specifically, the carrier concentration decreased from ≈10^20^ to 4 × 10^19^ cm^−3^ after annealing. Notably, despite the slight decrease in carrier concentration during annealing, a significant increase in charge carrier mobility is observed. This enhancement in charge carrier mobility leads to a notable improvement in charge carrier transport, which directly correlates with the substantial enhancement in the electrical performance of the material.

Consequently, the simultaneous enhancements in electrical conductivity and the Seebeck coefficient in Bi_2_Te_2.8_Se_0.2_S_0.01_ results in an exceptionally high PF, ≈51 μW cm^−1^ K^−2^, near room temperature, which is approximately 1.5 times larger than that of the sample produced through SPS. Interestingly, our polycrystalline Bi_2_Te_2.8_Se_0.2_S_0.01_ exhibits slightly higher PF than the n‐type single‐crystal Bi_2_Te_3_ material reported previously,^[^ ^44^ ^]^ due to the optimized Se/S doping composition. The largely improved PF can be attributed to two key factors. Firstly, the extrusion process facilitates grain growth and the formation of a more uniform microstructure with reduced defects and dislocations. This results in a reduction of grain boundary scattering, leading to enhanced charge carrier mobility. Additionally, the annealing process effectively reduces the concentration of defect density, further enhancing carrier mobility, which contributes to high S and σ simultaneously.

Figure 2g illustrates a significant increase in total thermal conductivity following the extrusion treatment, which continues to rise with subsequent annealing. Moreover, the observed upward trend in thermal conductivity at approximately 473 K is attributed to the intrinsic excitation of minority carriers. The total thermal conductivity encompasses contributions from the electrical component (*κ*_e_), lattice component (*κ*_L_), and bipolar component (*κ*_Bi_). The increased lattice thermal conductivity is associated with the reduced phonon scattering due to largely improved grain size and decrease of point defects. The combination of extrusion and annealing procedures results in a maximum zT value of approximately 1.12 at 348 K, representing a 21% increase compared to the sample produced through SPS (Figure 2i).

### 2.1 TE Device Cooling Performance

To construct TE modules in this study, commercial BiSbTe‐based *p*‐leg (Marlow Industries, Inc.) was employed as the p‐type material due to its similar electrical and thermal transport properties to the n‐type BiSeTe material. This selection helps avoid mechanical mismatches, such as differences in the coefficient of thermal expansion between the materials when cycling operation under large temperature gradients is desired.

**Figure 3:**
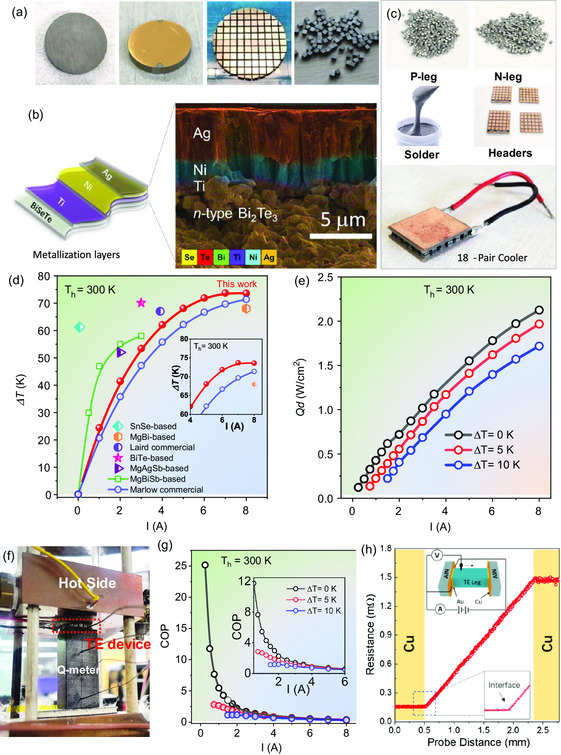
a) Device legs fabrication including metallization and cutting procedures. b) Characterization of metallized Ag–Ni–Ti layers on top of n‐type Bi_2_Te_2.8_Se_0.2_S_0.01_. c) TE modules assembly and full‐scale extruded Bi_2_Te_2.8_Se_0.2_S_0.01_ ‐based cooling modules. d) Measured Δ*T* as a function of the input electric current for an 18‐pair cooling module compared with state‐of‐art TE cooling devices.^[^ ^55^, ^56^, ^57^, ^58^ ^]^ e) Cooling power density of the 18‐pair module as a function of current. f) The measuring equipment and set‐up for cooling performance measurement. g) Coefficient of performance (COP) for the 18‐pair module working at various Δ*T* of 0, 5, and 10 K. h) Contact resistance measurement of the interfacial layers.

The fabrication process for high‐quality TE cooling modules is illustrated in **Figure** **3**a. The Bi_2_Te_2.8_Se_0.2_S_0.01_ wafers were diced at a low cutting speed to prevent cracking. The dimensions of the legs were cut into cubic shapes of 1.5 mm × 1.5 mm × 1.5 mm. To effectively translate the performance of the TE material to the device output, it is crucial to minimize parasitic losses caused by contact resistance between the TEG legs and electrodes. A device TE figure of merit zT_dev_ can be defined as:^[^ ^45^, ^46^, ^47^, ^48^ ^]^ ${\text{zT}}_{\text{dev}}={\text{zT}}_{m}\times \left(\frac{L}{L+2R_{c}\sigma }\right)$, where *L*, *R*_c_, *σ* and zT_m_ are the leg height, electrical contact resistance, electrical conductivity, and material zT of the legs, respectively. Ideally, if *r* = 0, then *zT*_dev_ = zT_m_. Hence, minimizing parasitic contact resistances is essential to maximize performance of the device.

In this study, a stack of multiple metal layers consisting of Ag/Ni/Ti/BiSeTe was designed to address issues related to coefficient of thermal expansion mismatch, mass diffusion, and contact resistance in the cooling module. To achieve uniform thickness for mass production, we employed magnetron sputtering to deposit three metallic layers onto the surface of the Bi_2_Te_2.8_Se_0.2_S_0.01_ wafer (Figure S9, Supporting Information). Subsequently, scanning electron microscopy (SEM) analysis confirmed the presence of a uniform contact layer with distinct boundaries between Ag/Ni/Ti/BiSeTe. The diffusion regions were quantified using energy dispersive X‐ray energy dispersive spectroscopy (Figure 3b). The thin diffusion layer promotes good chemical and thermal stability between BiSeTe and the barrier materials. The contact resistance of the n‐type leg was measured to be approximately <1 μΩ cm^2^ using the four‐probe method (Figure 3h), indicating successful design of metallic layers and soldering. The fabricated n‐type and p‐type legs were electrically connected in series and thermally connected in parallel to assemble various TE modules, e.g., cubic shape 18‐pair device, as shown in Figure 3c.

To assess the cooling performance of the 18‐pair TE module, the most effective approach is to measure the maximum temperature difference between its two sides under zero cooling loads. Figure 3f provides an overview of our measurement setup^[^ ^49^ ^]^ and the performance of our full‐scale Bi_2_Te_2.8_Se_0.2_S_0.01_‐BiSbTe cooling modules. When current passes through the module due to the Peltier effect, a temperature difference arises between the cold and hot sides. As the current increases with zero heat load, Δ*T* can ideally reach the maximum value of $\Delta T_{\text{max}}=\frac{1}{2}z_{d}T_{c}^{2}$,^[^ ^50^ ^]^ where *z*_d_ represents the *z* value of the device. The TE devices exhibited a record Δ*T*_max_ of up to 73.9 K with *T*_h_ = 300 K (Figure S10 and S11, Supporting Information). Notably, the maximum Δ*T* of our module surpasses that of a commercial Bi_2_Te_3_‐based module (purchased from Coherent, Inc., 31 pairs) tested under the same conditions.

The energy efficiency of a refrigeration system can be effectively evaluated using the coefficient of performance (COP), which is a crucial parameter for cooling modules. TE cooler requires electrical power (W) to drive heat flow from cold side (*Q*_C_) to hot side (*Q*_H_). COP is defined as the $\text{COP}=\frac{Q_{H}}{Q_{H}+W}$ *., W = P*_in_ *= VI,* $Q_{c}=kA\frac{dT}{dX}$, where *P*_in_ *, V, I, κ, A*, and $\frac{dT}{dX}$ are input power, applied voltage, current, thermal conductivity of Q‐meter, cross section of Q‐meter, and the slope of temperature gradient versus distance on the Q‐meter, respectively. Maximizing cooling efficiency requires the utilization of superior thermoelectric materials, minimizing temperature differences, and optimizing the applied current. Figure 3g illustrates the increase in the maximum COP from 1.2 (at Δ*T* = 10 K) to 2.9 (at Δ*T* = 5 K), with a maximum COP value reaching up to 25.2 under zero heat loading conditions.

To gain a deeper understanding of the cooling performance of the Bi_2_Te_2.8_Se_0.2_S_0.01_‐BiSbTe module, the cooling capacity (*Q*_c_), which represents the heat absorbed by the cold side of the module, was evaluated for 18‐pair modules. As depicted in Figure 3, the cooling heat flow increased with the current and gradually reached the maximum cooling density (*Q*_d_). With a small temperature difference (Δ*T*) between the cold (*T*_c_) and hot (*T*_h_) sides, the thermoelectric module exhibited a large cooling capacity due to reduced heat dissipation through thermal conduction in the materials.

Commercial solid‐state cooling devices typically utilize Bi_2_Te_3_‐based materials, achieving cooling power densities of up to ≈1.9 W cm^−2^ (based on leg lengths of ≈4.7 mm, KSM‐04007E, KELK Ltd.). However, such values are insufficient for advanced cooling applications, including electronic cooling, thermal management for laser devices, and hot spots, which require higher cooling power densities. As indicated in Equation (1), *Q*_d_ is primarily determined by the PF of the material. With its ultrahigh PF and respectable zT value near room temperature, Bi_2_Te_2.8_Se_0.2_S_0.01_ demonstrates enhanced performance compared to commercial Bi_2_Te_3_‐based TE devices in terms of low Δ*T* heat pumping.

The measured *Q*_d_ of the Bi_2_Te_2.8_Se_0.2_S_0.01_‐Bi_0.45_Sb_1.55_Te_3_ device at zero heat load was found to be 2.2 W cm^−2^ with a leg length of 1.5 mm, representing a 15.7% improvement compared to the commercial Bi_2_Te_3_‐based device (KSM‐04007E, KELK Ltd.). The maximum cooling power density of the Bi_2_Te_2.8_Se_0.2_S_0.01_‐BiSbTe device remains competitive with that of Bi_2_Te_3_‐based devices up to a Δ*T* of 10 K. By utilizing an improved p‐type material, even higher performance can be achieved at high Δ*T* in the future research.

## 3 Conclusion

In summary, we have successfully developed a high‐performance n‐type Bi_2_Te_2.8_Se_0.2_S_0.01_‐based thermoelectric material with exceptional electron mobility and power factor. This achievement was realized through the implementation of hot extrusion and annealing processes. The extrusion process plays a critical role in promoting grain growth, recrystallization, and the modulation of point defects. These factors collectively contribute to efficient charge carrier transport, resulting in a significant enhancement in thermoelectric power factor. Remarkably, the optimized n‐type Bi_2_Te_2.8_Se_0.2_S_0.01_ material exhibits a record‐breaking power factor value of ≈51 μW cm^−1^ K^−2^ around room temperature. Furthermore, we successfully fabricated full‐scale cooling modules based on the optimized n‐type Bi_2_Te_2.8_Se_0.2_S_0.01_ material. These modules demonstrated outstanding performance characteristics. Specifically, a maximum temperature difference of 73.9 K with a maximum cooling density of 2.2 W cm^2^ was achieved with a hot‐side temperature of 300 K. These results surpass the performance of commercially available Bi_2_Te_3_‐based thermoelectric devices. The presented processing techniques are easily implementable and lead to potential for application to other thermoelectric materials, particularly chalcogenide compounds with similar eutectic phase transformation.

## Conflict of Interest

The authors declare no conflict of interest.

## Supporting information

Supplementary Material

## Data Availability

The data that support the findings of this study are available from the corresponding author upon reasonable request.
