# Supplementary material for: Thermoelectric Cooling Performance Enhancement in BiSeTe Alloy by Microstructure Modulation via Hot Extrusion
Source: Small Sci. 2023 Dec 27;4(2):2300245. doi: 10.1002/smsc.202300245 (PMC11935137; doi:10.1002/smsc.202300245)
Supplement: Supplementary file 1 — Supplementary Material [file SMSC-4-2300245-s001.pdf]

Supporting Information

Thermoelectric Cooling Performance Enhancement in BiSeTe Alloy by Microstructure Modulation via Hot Extrusion

Yu Zhang[1], Guang Xu[1], Amin Nozariasbmarz[1], Wenjie Li[1], Lavanya Raman[1], Congcong Xing[1], Shweta Sharma[1], Na Liu[1], Subrata Ghosh[1], Giri Joshi[2], Mohan Sanghadasa[3], Priya Shashank, [1] Bed Poudel[1]**

[1]Department of Materials Science and Engineering, Pennsylvania State University, University Park, Pennsylvania 16802, United States

[2]Nanohmics Inc, 6201 E Oltorf St, Austin, TX 78741

[3]U.S. Army Combat Capabilities Development Command Aviation & Missile Center, Redstone Arsenal, AL, 35898 USA

**Corresponding author. Email: yvz5897@psu.edu (Y.Z.); bup346@psu.edu (B.P.).*

Materials and Methods

Materials and Synthesis

Bismuth (99.9%, pieces), antimony shots (Sb, Alfa Aesar 99.9999%), selenium shots (Se, Sigma Aldrich 99.9%), tellurium lumps (Te, Alfa Aesar 99.9 %) and sulfur flasks (S, Sigma Aldrich, 99.9%) were used as received, without further purification.

To synthesize 20g $Bi_2Te_{2.8}Se_{0.2}S_{0.01}$ powder, high-purity elements of Bi (99.9%, pieces), Te (99.9%, lumps), Se (99.9%, pellets) and S (99.9%, flakes) were loaded into a hardened steel vial along with steel balls with ball to powder ratio of 1:1 and ball milled for 13 hours using SPEX mixer/mill (Model 8000D, SPEX SamplePrep) and clamp speed of 1060 cycles per minute to obtain homogeneous powder. The powder was firstly compacted under room temperature, then loaded into a metal die and extruded at temperatures range of 320 °C to 450 °C under pressure of 200-600 MPa, producing cylindrical rods with 12.5 mm diameter as shown in Figure 1a-b. Subsequently, the obtained rod was thermally annealed at 500 °C for 10 min in Ar atmosphere. For comparison, the ball milled $Bi_2Se_{0.2}Te_{2.8}$ powdered alloys were also directly consolidated by spark plasma sintering (SPS, Dr. Sinter-625V, Fuji, Japan) at 500 °C under a pressure of 40 MPa for 5 minutes.

Materials characterization

The electrical conductivity and Seebeck coefficient were measured simultaneously (ULVAC-RIKO ZEM-3 system, Japan) using 2 mm × 2 mm × 12 mm bar. We estimate an error of ca. 5% in the measurement of both electrical conductivity and the Seebeck coefficient. Temperature-dependent thermal properties were determined by measuring thermal diffusivity with a laser flash system (LFA-467 HT HyperFlash®, Germany). Specific heat was measured on a differential scanning calorimeter (Netzsch DSC 214, Germany, heating/cooling rate of 15 K/min). The thermal conductivity, κ_{total}, was calculated from $\kappa_{total} = D \times \rho \times Cp$, where D, ρ, and Cp are thermal diffusivity, density, and specific heat, respectively. The density is measured using the Archimedes method. The estimated error in thermal conductivity measurement is estimated to be about ±4%. Electronic thermal conductivity (κ_e) of $Bi_2Se_{0.2}Te_{2.8}S_{0.01}$ is calculated from Wiedemann-Franz law, $\kappa_e = L\sigma T$, where L and σ, are Lorenz number and electrical conductivity. The Lorenz number is calculated based on the measured Seebeck coefficient: $L=[1.5+exp(-|S|/(116\ \mu VK^{-1}))]\ 10^{-8}\ W\Omega K^{-}$

[2]. The results presented here are an average of the results obtained after measuring 3 pellets produced under identical conditions. Measurements between different samples have standard deviations below 10%. The microstructures were characterized by field emission scanning electron microscopy (FEI Verios G4), energy dispersive spectroscopy (EDS, Oxford Aztec), and EBSD (FEI Apero S). X-ray diffraction analysis was carried out on a PANalytical Empyrean with Cu-K_α radiation in 2θ angle range of 10-60°. Charger carrier density and mobility were characterized by LakeShore Hall Effect System (8400 Series HMS, LakeShore) using a magnetic field of 0.9 T. The custom-built scanning probe measurement system was used to analyze the contact resistance.

Module Fabrication

The obtained bulk pellet was polished and cleaned thoroughly by ultrasonic stirring. Afterward, a magnetron sputtering deposition was applied to sputter three metallic layers of Ti/Ni/Ag on each side of the pellets. The pellet was then diced along the press direction into a certain dimension leg (1.5 mm (length) × 1.5 mm (width) × 1.5 mm (height)) using a wire saw. The legs with preprocessed barrier layers are welded on substrates by Sn/Pb solder to form series circuits of thermoelectric legs.

Heat flow measurement

The heat flow (Q) was measured using a Q-meter, which is a standard material (copper, brass, or graphite are commonly used) with a cylindrical or rectangular shape connected to the heat sink. The temperature gradient along the Q-meter (graphite were used for cooling measurement in this work) was measured by using four thermocouples located at specific distances. Then Q can be calculated by $Q = \kappa \cdot A \cdot \frac{dT}{dx}$, where κ, A, and dT/dx are the thermal conductivity, cross-sectional area, and the slope of temperature difference versus distance on the Q-meter.

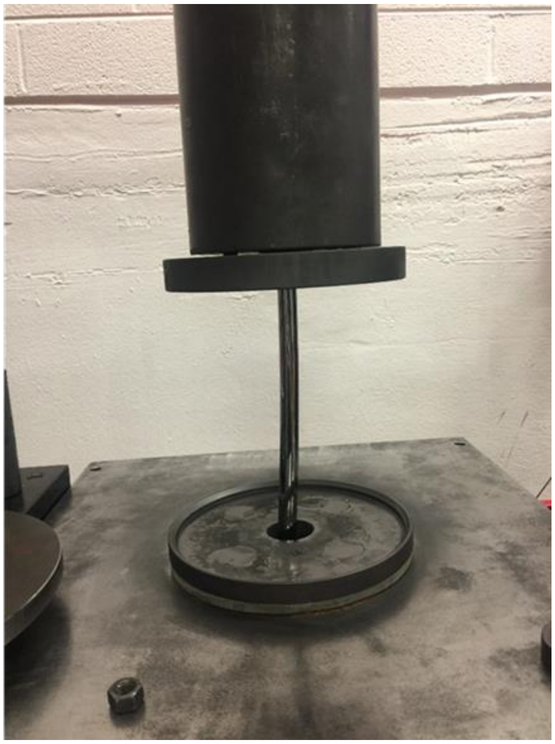

Figure S1. General appearance of the extruded rod (12.5 mm diameter rod) from a metallic die.

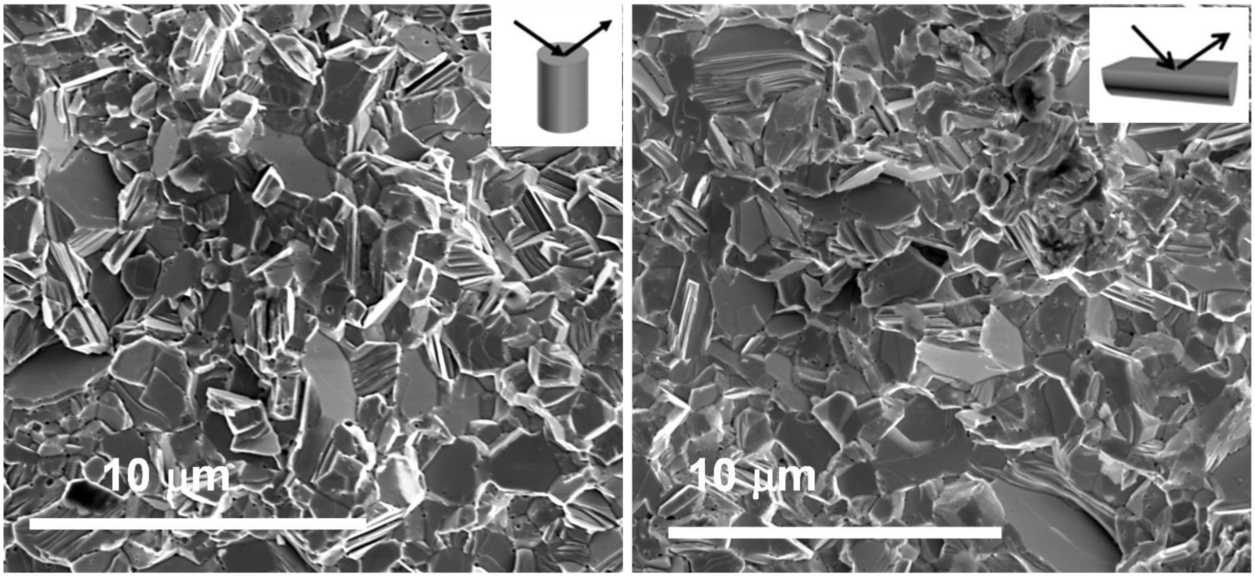

Figure S2. Representative (a) top view and (b) cross-section SEM micrograph of grain structure of samples consolidated by SPS only.

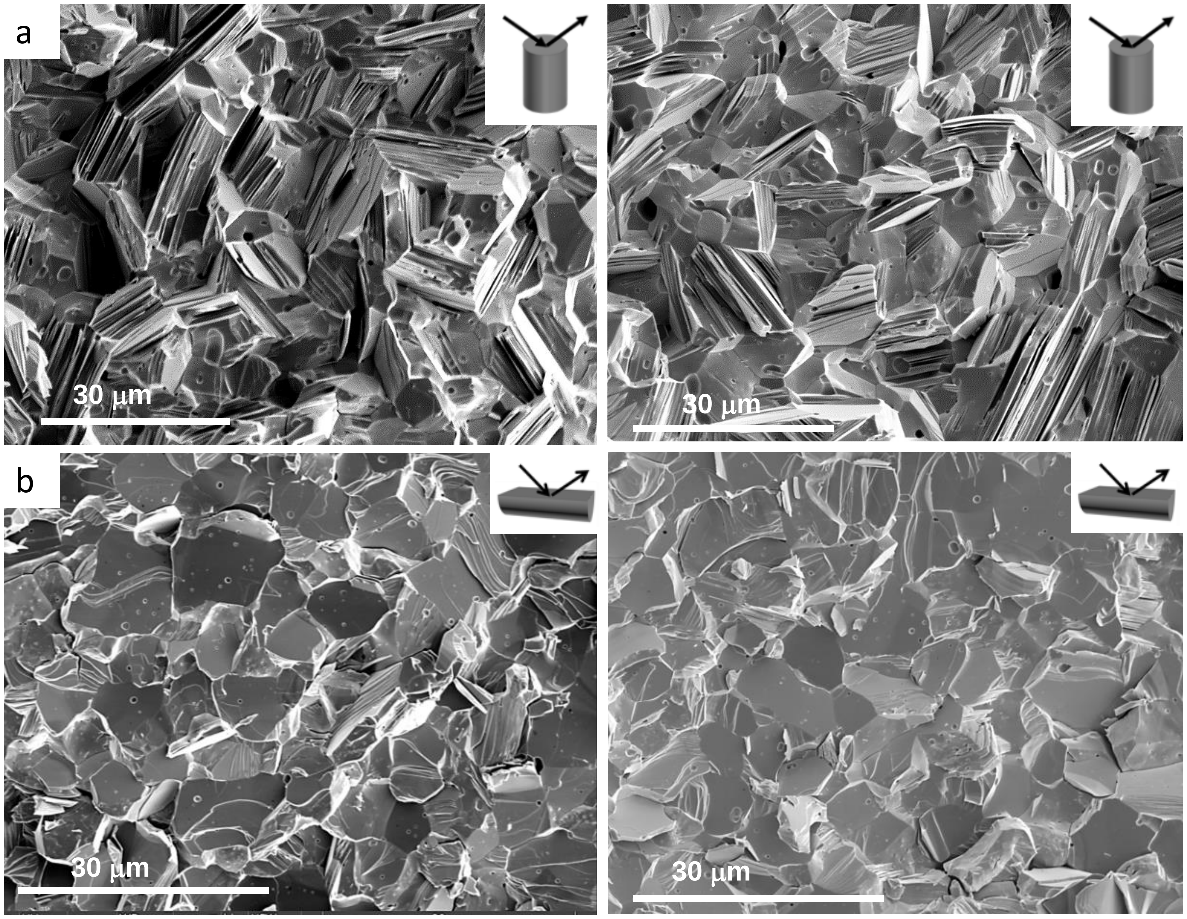

Figure S3. Representative (a) top view and (b) cross-section SEM micrograph of grain structure of samples consolidated by hot extrusion.

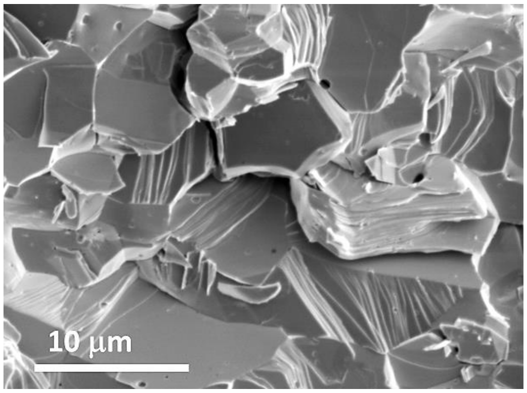

Figure S4. Representative (a) top view and (b) cross-section SEM micrograph of grain structure of samples consolidated by “Extrusion + Annealing”.

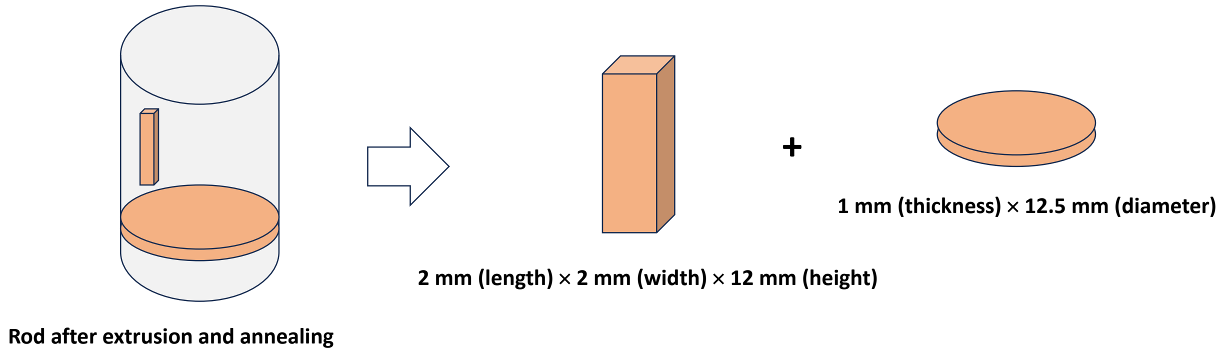

Figure S5. $Bi_2Te_{2.8}Se_{0.2}S_{0.01}$ pellet sample cutting directions and dimensions for TE properties measurement.

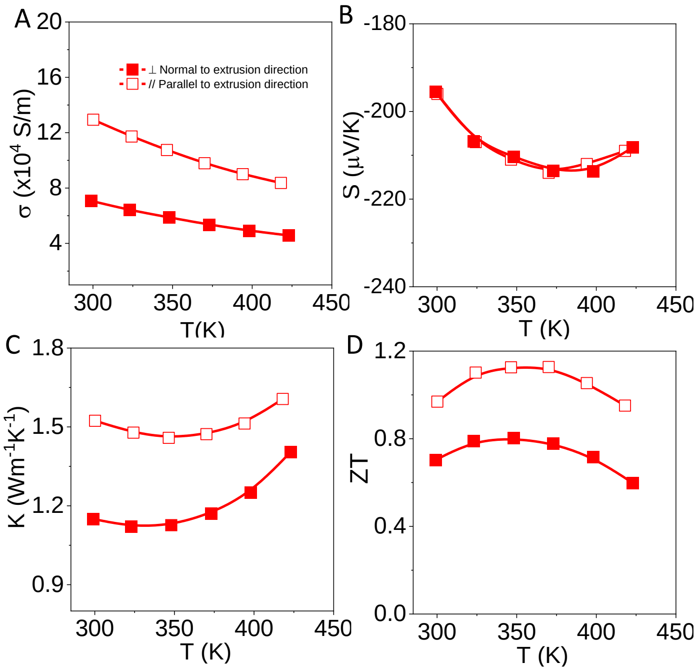

Figure S6. Temperature dependence of (A) electrical conductivity, σ; (B) Seebeck coefficient, S; (C) total thermal conductivity, κ and (D) TE Figure of merit, ZT of a $Bi_2Te_{2.8}Se_{0.2}S_{0.01}$ pellet measured in two directions: parallel to extrusion direction (//) and normal to extrusion direction (⊥).

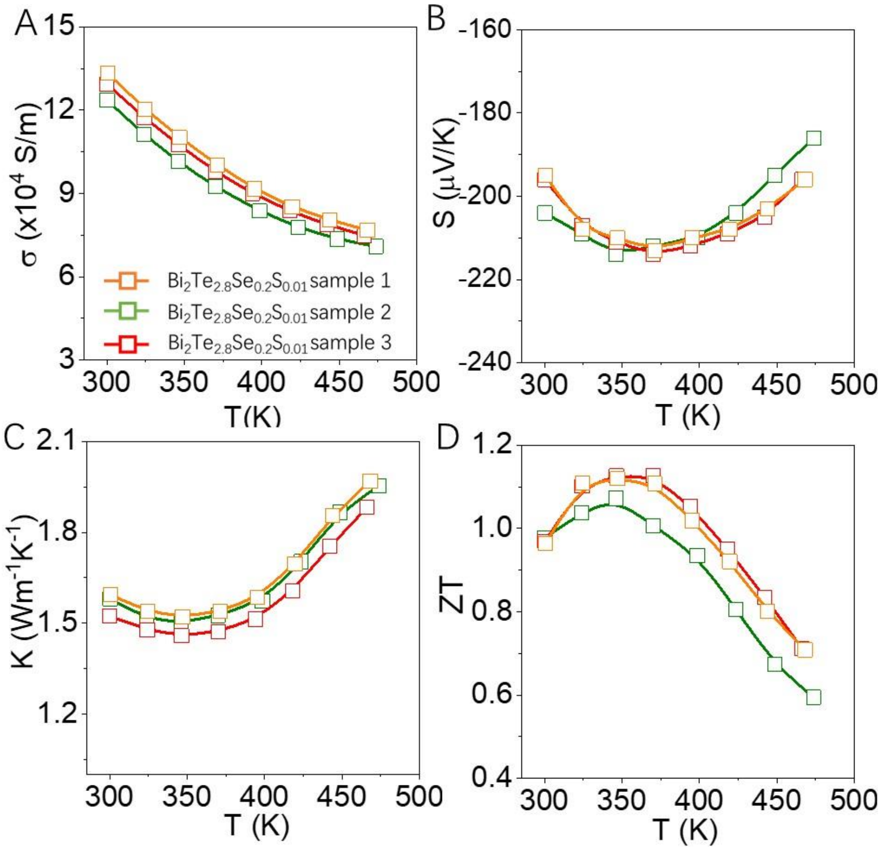

Figure S7. Reproducibility of TE properties of three $Bi_2Te_{2.8}Se_{0.2}S_{0.01}$ samples among different batches synthesized separately. Temperature dependent TE performance of (A) electrical conductivity (σ), (B) Seebeck coefficient (S), (C) total thermal conductivity (κ) and (D) TE figure of merit (zT).

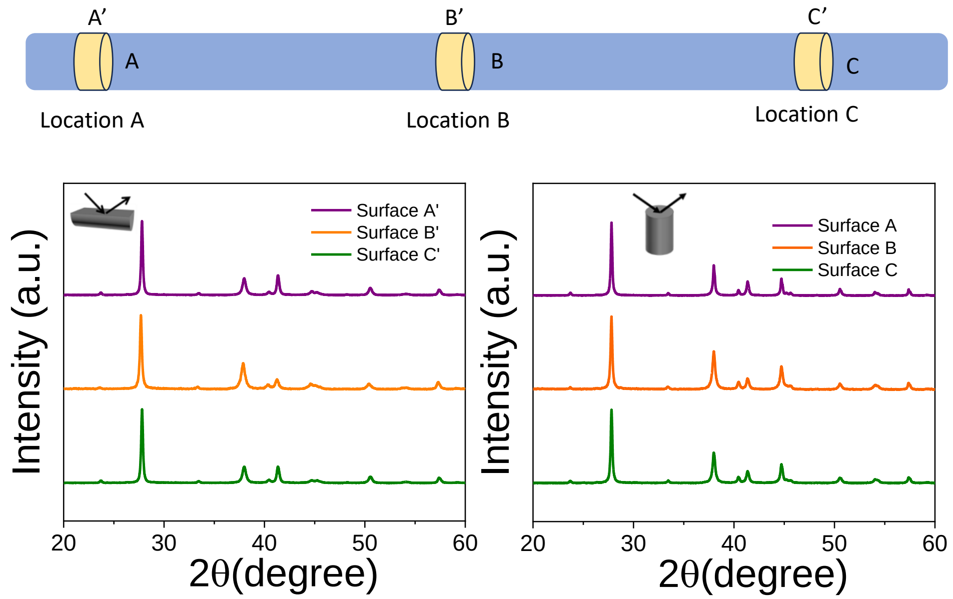

Figure S8. Uniformity of hot extruded rods on three independent locations.

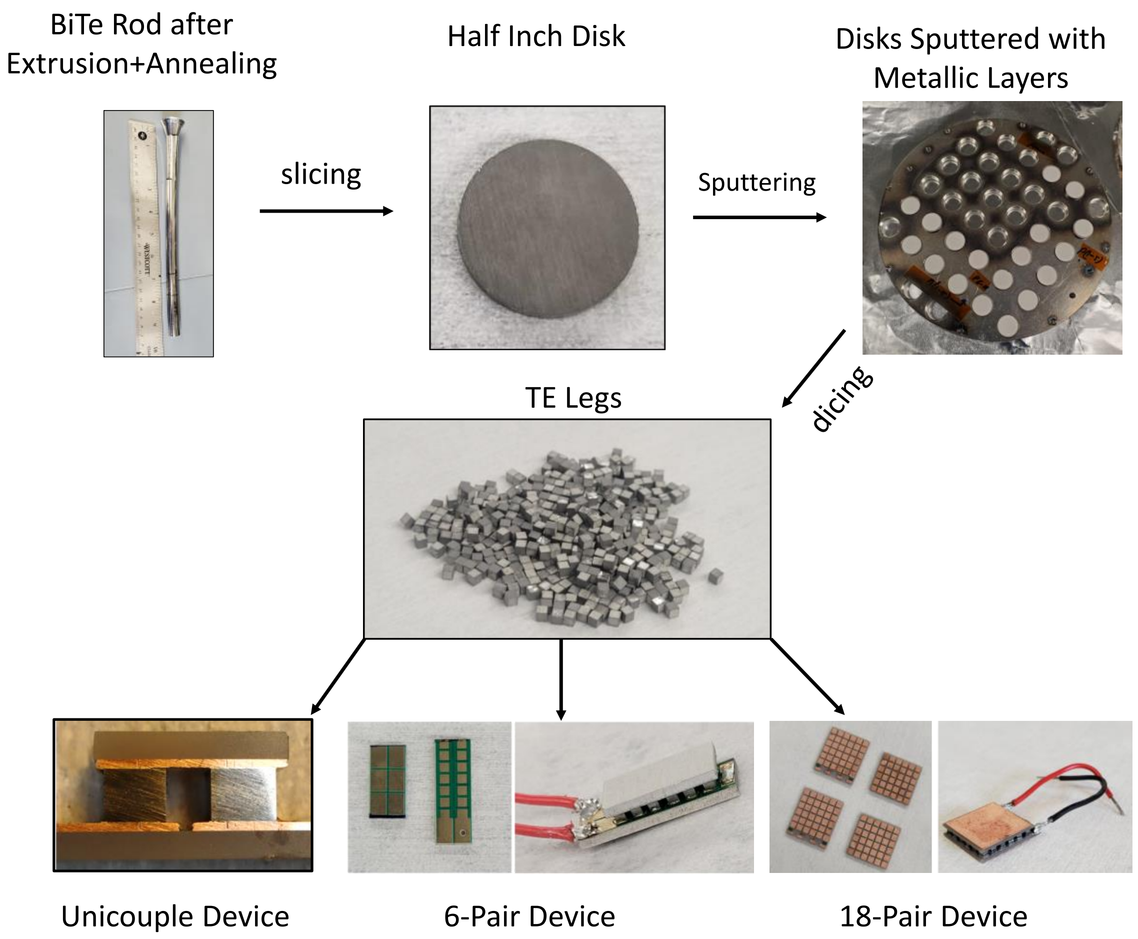

Figure S9. TE cooling device fabrication includes metallization and cutting procedures prepared from extruded rod.

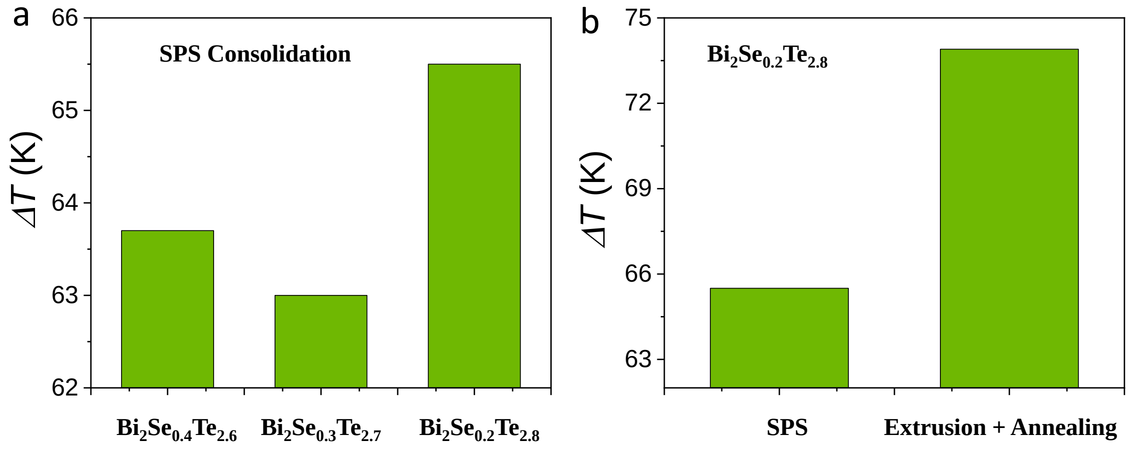

Figure S10. Measured ΔT as a function of the composition of $Bi_2Te_{2-x}Se_xS_{0.01}$ (0.2≤X≤0.4). (a) Samples consolidated solely by SPS, (b) $Bi_2Te_{2.8}Se_{0.2}S_{0.01}$ processed by SPS and combined procedures.

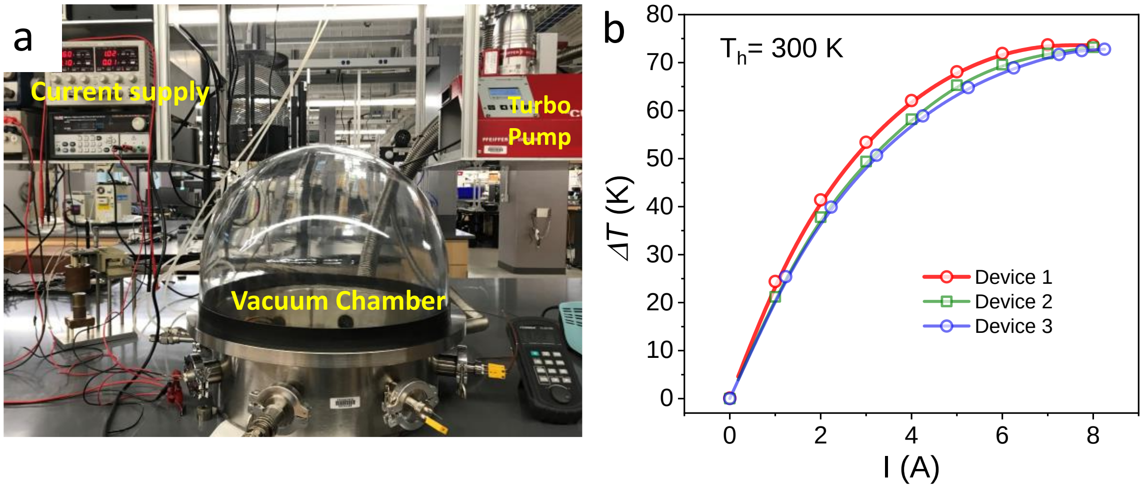

Figure S11. (a) Custom-made TE cooling device performance testing system. (b) Repeatability Test: measured ΔT as a function of the input electric current for three 18-pair cooling modules.

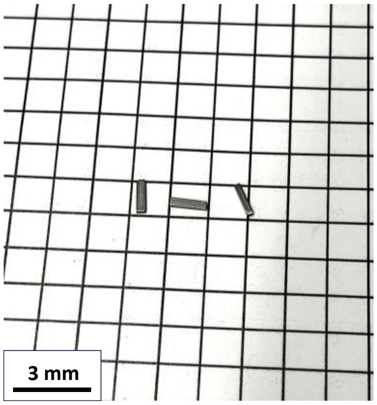

Figure S12. Micro TE dices with cross-section size of 400 μm × 300 μm prepared from the $Bi_2Te_{2.8}Se_{0.2}S_{0.01}$ extrusion sample.
